# Effects of low acid-binding capacity specialty soy protein sources on nursery pig performance in a commercial environment

**DOI:** 10.1093/tas/txae180

**Published:** 2024-12-23

**Authors:** Ethan B Stas, Alan J Warner, Zach B Post, Chad W Hastad, Jamil E G Faccin, Mike D Tokach, Jason C Woodworth, Joel M DeRouchey, Robert D Goodband, Jordan T Gebhardt

**Affiliations:** Department of Animal Sciences and Industry, Kansas State University, Manhattan, KS 66506-0201, USA; New Fashion Pork, Jackson, MN 56143-1601, USA; New Fashion Pork, Jackson, MN 56143-1601, USA; New Fashion Pork, Jackson, MN 56143-1601, USA; Department of Animal Sciences and Industry, Kansas State University, Manhattan, KS 66506-0201, USA; Department of Animal Sciences and Industry, Kansas State University, Manhattan, KS 66506-0201, USA; Department of Animal Sciences and Industry, Kansas State University, Manhattan, KS 66506-0201, USA; Department of Animal Sciences and Industry, Kansas State University, Manhattan, KS 66506-0201, USA; Department of Animal Sciences and Industry, Kansas State University, Manhattan, KS 66506-0201, USA; Department of Diagnostic Medicine/Pathobiology, College of Veterinary Medicine, Kansas State University, Manhattan, KS 66506-0201, USA

**Keywords:** acid-binding capacity, nursery pig, poultry meal, specialty soy protein, spray-dried blood plasma, zinc

## Abstract

Two experiments were conducted to determine the effects of low acid-binding capacity (**ABC**) specialty soy protein sources on weanling pig performance. In experiment 1, 2,260 pigs, initially weighed 6.7 kg, were used to determine the effects of low ABC soy proteins as a replacement to poultry meal (**PM**) or spray-dried blood plasma (**SDBP**). Treatments were arranged in a 2 × 2 factorial plus a control diet fed in two phases. There were 20 or 21 pigs per pen with 22 replications (pens) per treatment. The control diet contained PM (AV-E Digest, XFE Products, Des Moines, IA) and SDBP (Appetein, APC Inc., Ankeny, IA). Specialty soy protein concentrate (**SSPC**; AX3 Digest, Protekta, Newport Beach, CA) and microbial-enhanced soybean meal (**MESBM**; MEPRO; Prairie Aquatech, Brookings, SD) were used to replace PM or PM and SDBP on a standardized ileal digestible Lys basis. From d 0 to 21 and d 0 to 42, pigs fed either soy protein source replacing PM had greater (*P *≤ 0.016) average daily gain (**ADG**) and average daily feed intake than pigs fed PM. From d 0 to 21, pigs fed SSPC had increased (*P *< 0.001) G:F compared with pigs fed MESBM and those fed either soy protein source replacing SDBP had increased (*P *= 0.044) G:F compared with pigs fed SDBP. In experiment 2, 1,057 pigs, initially weighed 6.2 kg, were used to determine the effects of diet ABC at a pH of 4 (**ABC-4**) with specialty soy proteins with or without pharmacological levels of Zn from ZnO. Experimental diets were fed in two phases with 22 pigs per pen and 12 replications per treatment. Dietary treatments were arranged in a 2 × 2 factorial with main effects of ABC-4 (low or high) and pharmacological levels of Zn from ZnO (105 or 2,000 mg/kg). The low ABC-4 diet without ZnO was formulated to 150 and 200 meq/kg using SSPC in phases 1 and 2, respectively. The high ABC-4 diet used enzymatically treated soybean meal (HP 300, Hamlet Protein, Findlay, OH) which increased the ABC-4 by 127 and 104 meq/kg in phases 1 and 2, respectively. From d 0 to 21 and d 0 to 42, there was an ABC-4 × ZnO interaction (*P *≤ 0.026) observed where pigs fed low ABC-4 diets had greater (*P *< 0.05) ADG and G:F than pigs fed high ABC-4 diets without ZnO, but when diets contained added ZnO, there were no differences based on ABC-4. In conclusion, low ABC specialty soy proteins can be used to achieve low dietary ABC-4 levels to improve the performance of weanling pigs and provide a similar response to those fed pharmacological levels of Zn.

## Teaser

Acid-binding capacity of the diet can be lowered with specialty soy protein sources which offers an alternative to pharmacological levels of Zn in weanling pig diets.

## INTRODUCTION

At the time of weaning, pigs have an underdeveloped gastrointestinal tract and their stomach hydrochloric acid secretion is limited until approximately 7 to 8 weeks of age (Pluske, 2016). This can lead to an elevated stomach pH that will negatively affect protein digestion even with the presence of high-quality protein sources (Yen, 2001). Certain ingredients common in early nursery diets possess high acid-binding capacity (**ABC**) characteristics that can raise stomach pH and exacerbate this situation (Batonon-Alavo et al., 2016). Incorporating low acid-binding ingredients has been suggested as a method to maintain an acidic gastric environment to improve the growth and health status of newly weaned pigs (Lawlor et al., 2005). However, few studies have evaluated the effect that low acid-binding ingredients have on weanling pig performance and health status.

Soybean meal is the predominant protein source used in swine diets, but it contains antinutritional factors that cause a hypersensitive immune response in the gastrointestinal tract of newly weaned pigs (Li et al., 1990). This can result in increased occurrence of post-weaning diarrhea and reduced growth rate. As a result, the addition of soybean meal to the diet is limited immediately after weaning. High-quality specialty protein sources, such as animal proteins or vegetable proteins with minimal antinutritional factors, are used as partial replacements for soybean meal to meet the pig’s amino acid (**AA**) requirements. Spray-dried blood plasma (**SDBP**) has been observed to promote growth, immune status, and gut health of newly weaned pigs (Balan et al., 2021; Kim et al., 2022). Poultry meal (**PM**) is a lower-cost animal protein source and has been observed to have similar performance as high-quality protein sources such as SDBP (Zier et al., 2004; Frame et al., 2021). However, these animal protein sources have been observed to have a high ABC at a pH of 4 (ABC-4) compared with certain vegetable protein sources (Stas et al., 2022). Vegetable proteins such as further-processed soy proteins can provide an excellent AA profile with reduced antinutritional factors (Deng et al., 2023; Jang and Kim, 2023). Further-processed soy proteins are associated with lower levels of glycinin and β-conglycinin that cause villus atrophy and crypt hyperplasia in the small intestine of young pigs (Kim et al., 2010). Specialty soy proteins have been observed to vary in their ABC-4 value which could have a large impact on weanling pig stomach pH (Stas et al., 2022). Incorporating low ABC-4 soy proteins may offer an added benefit not previously realized and, thus, could serve as a replacement for PM and SDBP.

In addition to specialty protein sources, pharmacological levels of Zn from ZnO are commonly used to decrease the prevalence of post-weaning diarrhea and improve the performance of weanling pigs (Bonetti et al., 2021). However, the use of ZnO at pharmacological levels has raised concerns about antimicrobial resistance and heavy metal accumulation in the environment (Ciesinski et al., 2018; Ma et al., 2021). Therefore, other dietary strategies are being explored to maintain performance and fecal integrity in low Zn diets. Low ABC specialty protein sources may be used to formulate to low diet ABC-4 levels and maintain performance and fecal consistency similar to pharmacological levels of Zn from ZnO.

We hypothesized that low ABC-4 specialty soy protein sources can serve as alternatives for more expensive, high ABC-4 animal proteins and using these soy protein sources in a low ABC-4 diet formulation strategy might be an alternative to pharmacological concentrations of Zn from ZnO.

## MATERIALS AND METHODS

### General

The Kansas State University Institutional Animal Care and Use Committee approved the protocols used in these experiments. Both experiments were conducted at commercial research sites owned and operated by New Fashion Pork, Jackson, MN. Experiment 1 was conducted in two commercial research sites. The first nursery site consisted of a single nursery room, whereas the second nursery site used two identical rooms. Experiment 2 was conducted in a single room at a commercial research site. All nursery facilities are completely enclosed, environmentally controlled, and mechanically ventilated to maintain a room temperature between 18 to 32 °C for weight ranges of 5 to 30 kg.

#### ABC-4 analysis.

For experiment 1, samples of PM (AV-E Digest, XFE Products, Des Moines, IA), SDBP (Appetein, APC Inc., Ankeny, IA), specialty soy protein concentrate (SSPC; AX3 Digest; Protekta; Newport Beach, CA), and microbially enhanced soybean meal (MEPRO; Prairie Aquatech; Brookings, SD) were analyzed for ABC-4. Calculated ABC-4 values of experimental diets were determined using the analyzed ABC-4 values of experimental protein sources. All other ingredient ABC-4 values were obtained from Stas et al. (2024). The complete diets for experiment 1 were also analyzed for ABC-4. For experiment 2, a sample of each ingredient used in diet formulation and the complete diet were analyzed for ABC-4. All samples were analyzed at the Kansas State University Swine Nutrition Laboratory in Manhattan, KS (Table 1). ABC analysis used the procedure outlined by Stas et al. (2022). Half a gram of each sample was suspended in 50 mL of distiller-deionized water using a magnetic stir bar and stir plate. Titrations were performed by the addition of 0.1 N HCl in increments of 0.1 to 5.0 mL depending on the sample and stage of titration. Once a stable pH of 4.00 ± 0.04 was reached, ABC-4 is calculated as the amount of acid in milliequivalents (meq) required to achieve a pH of 4 for 1 kg of sample. Each diet and ingredient sample were analyzed in triplicate and the average was used for the overall ABC-4 value. In addition, water samples were collected from the nipple waterer of 3 randomly selected pens for the pH measurement of the drinking water.

**Table 1.** Analyzed ABC at a pH of 4 (ABC-4) of ingredients used in experimental diets^*a*^

Item	Initial pH	ABC-4 (meq/kg)
Ingredients, %		
Corn	6.38	84
Soybean meal (46.8% CP)	7.05	602
Cereal blend	6.08	107
Poultry meal^*b*^	6.74	1,007
Spray-dried blood plasma^*c*^	6.99	713
Specialty soy protein concentrate^*d*^	3.95	-13
Microbially enhanced soybean meal^*e*^	5.01	107
Enzymatically treated soybean meal^*f*^	6.29	753
Oat groats	6.44	107
Crystalline lactose	7.09	53
Fat blend	6.60	363
Beef tallow	4.90	16
Limestone	9.68	18,384
Dicalcium phosphate (19% P)	7.03	3,673
Salt	5.24	10
L-Lys	5.80	80
DL-Met	6.13	140
L-Thr	5.54	160
L-Trp	5.11	120
L-Val	5.53	193
L-Ile	5.76	200
Choline chloride	5.21	40
Zinc oxide	9.88	21,863
Organic Zn (21.0%)^*g*^	7.14	12,820
Vitamin-trace mineral premix	4.67	1,727
Organic Mn (20.0%)^*g*^	8.22	2,347
Fumaric acid	2.34	-10,862

^*a*^ABC-4 was determined by the amount of 0.1 N HCl required to lower the initial pH of a sample to a stable pH of 4.00 ± 0.04. Samples with a pH less than 4 followed the same procedure but were titrated with NaOH to raise the initial pH. Each sample was analyzed in triplicate. Only experimental protein sources were analyzed for experiment 1, whereas all ingredients included in experimental diets were analyzed for experiment 2.

^*b*^ AV-E Digest, XFE Products, Des Moines, IA.

^*c*^ Appetein, APC Inc., Ankeny, IA.

^*d*^ AX3 Digest, Protekta, Newport Beach, CA.

^*e*^MEPRO, Prairie Aquatech, Brookings, SD.

^*f*^HP 300, Hamlet Protein, Findlay, OH.

^*g*^ Zinpro, Eden Prairie, MN.

#### Chemical analysis.

Diet samples for both experiments were collected from each feeder using a feed probe to obtain a representative sample for each diet and phase. Diet samples were stored at −20 °C until they were homogenized, subsampled, and submitted for analysis. Experimental protein sources were formulated according to manufacturer-supplied nutrient specifications and standardized ileal digestible (**SID**) coefficients of crude protein (**CP**) and AAs (Table 2). A sample of PM, SDBP, SSPC, MESBM, and enzymatically treated soybean meal (**ESBM**; HP 300; Hamlet Protein; Findlay, OH) was also collected for analysis to confirm dietary formulation. All ingredient and diet samples were submitted to the Agricultural Experiment Station Chemical Laboratories (University of Missouri-Columbia, Columbia, MO) for analysis of CP (method 990.03; AOAC International, 2006), ash, dry matter (**DM**; method 935.29; AOAC International, 2006), ether extract (**EE**) (ANKOM Technology, 2004), and crude fiber (ANKOM Technology, 2005).

**Table 2.** Manufacturer-provided nutrients specification of protein sources used in diet formulation (as-fed basis)^*a*^

Item	Poultry meal^*b*^	Spray-dried blood plasma^*c*^	Specialty soy protein concentrate^*d*^	Microbially enhanced soybean meal^*e*^	Enzymatically treated soybean meal^*f*^
Item, %					
DM	93.90	92.00	92.00	95.50	93.00
CP	51.35	77.85	68.00	70.60	56.00
EE	16.05	2.00	—	0.85	3.00
Crude fiber	1.30	—	4.00	5.40	4.00
Ash	13.70	8.70	3.60	1.30	7.00
Amino acids					
Arg	3.73	4.39	4.90	4.47	3.97
Cys	0.99	2.60	1.00	1.13	0.82
His	1.29	2.53	1.70	1.74	1.47
Ile	2.04	2.69	3.20	3.57	2.75
Leu	3.61	7.39	5.40	5.71	4.32
Lys	3.02	6.90	4.10	4.12	3.45
Met	0.89	0.79	1.00	1.02	0.77
Phe	2.44	4.25	3.70	3.70	2.90
Thr	2.04	4.47	2.70	2.78	2.18
Trp	0.44	1.41	0.90	1.00	0.77
Val	2.52	5.12	3.40	3.62	2.82
Analyzed values, %^*g*^					
DM	90.11	88.68	93.97	94.53	93.74
CP	48.38	76.21	64.94	66.59	53.53
EE	12.76	—	0.02	—	1.12
Crude fiber	1.45	—	6.02	4.75	4.71
Ash	14.58	8.04	2.94	1.35	7.11

^*a*^Nutrient specifications of each protein source used in diet formulation. Values were obtained from manufacturer of each protein source.

^*b*^AV-E Digest, XFE Products, Des Moines, IA.

^*c*^Appetein, APC Inc., Ankeny, IA.

^*d*^AX3 Digest, Protekta, Newport Beach, CA.

^*e*^MEPRO, Prairie Aquatech, Brookings, SD.

^*f*^HP 300, Hamlet Protein, Findlay, OH.

^*g*^A sample of each soy protein source was submitted to the Agricultural Experiment Station Chemical Laboratories (University of Missouri-Columbia, Columbia, MO).

### Experiment 1

#### Animals and treatment structure.

The objective of experiment 1 was to evaluate two low ABC-4 specialty soy protein sources as a replacement to PM or SDBP in weanling pig diets. A total of 2,260 pigs (PIC TR4 × [Fast LW × PIC L02], initially weighed 6.7 kg) were used in a 42-d trial across two facilities. In the first facility, there were 20 pigs per pen and 10 replications per treatment spread across two rooms. In the second facility, there were 21 pigs per pen and 12 replications per treatment. In total, there were 22 replications per treatment. Pigs were weaned at approximately 21 d of age and pens were randomly assigned to one of the five dietary treatments based on initial body weight (**BW**).

Five dietary treatments were arranged in a 2 × 2 factorial plus a control diet fed in two phases (Tables 3 and 4). The control diet contained 9.5% PM in phases 1 and 2 and 4.13% (phase 1) or 2.75% (phase 2) SDBP. The SDBP utilized was of bovine origin. The other four diets included SSPC or MESBM replacing PM or PM and SDBP in the control diet. Protein sources were added to the diet to maintain a similar level of soybean meal and balanced for SID AAs by using feed-grade AAs. Thus, soy protein sources were added to the diet at 6% and 13% (phase 1) or 6% and 10.75% (phase 2) to replace PM or PM and SDBP, respectively. Dietary treatment structure allowed the opportunity to compare each specialty soy protein source to each other, the soy protein sources as a replacement of PM, and the soy protein sources as a replacement of SDBP.

**Table 3.** Phase 1 diet composition (as-fed basis), experiment 1^*a*^

		Specialty soy protein concentrate^*b*^		Microbially enhanced soybean meal^*c*^	
Item	Control	Replacing poultry meal	Replacing poultry meal and blood plasma	Replacing poultry meal	Replacing poultry meal and blood plasma
Ingredients, %					
Corn	44.10	46.34	43.36	46.17	42.96
Soybean meal (46.8% CP)	18.59	18.58	18.54	18.74	18.87
Cereal blend	13.10	13.10	13.10	13.10	13.10
Poultry meal^*d*^	9.50	—	—	—	—
Specialty soy protein conentrate^*b*^	—	6.00	13.00	—	—
Microbially enhanced soybean meal^*c*^	—	—	—	6.00	13.00
Spray-dried blood plasma^*e*^	4.13	4.13	—	4.13	—
Oat groats	3.75	3.75	3.75	3.75	3.75
Crystalline lactose	1.38	1.38	1.38	1.38	1.38
Fat blend	2.00	2.00	2.00	2.00	2.00
Beef tallow	0.50	0.50	0.50	0.50	0.50
Limestone	0.38	0.60	0.33	0.68	0.53
Dicalcium phosphate (19% P)	0.23	1.05	1.40	0.95	1.15
Salt	—	0.25	0.45	0.33	0.63
L-Lys	0.42	0.42	0.42	0.42	0.42
DL-Met	0.20	0.21	0.22	0.20	0.20
L-Thr	0.26	0.27	0.26	0.26	0.25
L-Trp	0.08	0.07	0.07	0.06	0.05
L-Val	0.11	0.10	0.06	0.10	0.05
L-Ile	0.11	0.09	—	0.08	—
Copper chloride	0.04	0.04	0.04	0.04	0.04
Choline chloride	0.03	0.03	0.03	0.03	0.03
Zinc oxide	0.30	0.30	0.30	0.30	0.30
Vitamin E	0.05	0.05	0.05	0.05	0.05
Vitamin-trace mineral premix^*f*^	0.25	0.25	0.25	0.25	0.25
Benzoic acid	0.50	0.50	0.50	0.50	0.50
Organic Mn (20.0%)^*g*^	0.02	0.02	0.02	0.02	0.02
Total	100	100	100	100	100

Item	Control	Specialty soy protein concentrate^*b*^		Microbially enhanced soybean meal^*c*^	
		Replacing poultry meal	Replacing poultry meal and blood plasma	Replacing poultry meal	Replacing poultry meal and blood plasma
SID amino acids, %					
Lys	1.36	1.36	1.36	1.36	1.36
Ile:Lys	59	59	61	60	63
Leu:Lys	105	108	113	109	114
Met:Lys	33	33	36	33	35
Met and Cys:Lys	56	56	56	56	56
Thr:Lys	66	66	66	66	66
Trp:Lys	22.2	22.2	22.3	22.1	22.2
Val:Lys	70	70	70	70	70
His:Lys	34	35	36	35	36
Total Lys, %	1.55	1.52	1.51	1.52	1.52
NE NRC, kcal/kg	2,330	2,410	2,381	2,401	2,361
SID Lys:NE, g/Mcal	5.84	5.65	5.71	5.67	5.76
CP, %	22.0	21.3	22.6	21.5	23.0
Ca, %	0.59	0.59	0.59	0.59	0.59
P, %	0.55	0.58	0.62	0.55	0.59
STTD P, %	0.43	0.43	0.43	0.43	0.43
ABC-4, meq/kg^*h*^	445	412	336	432	385
Analyzed values, %					
DM	89.09	89.34	89.12	89.57	90.00
CP	19.98	21.99	22.18	22.27	22.34
Crude fat	4.14	3.70	3.49	3.17	3.13
Crude fiber	2.10	2.06	2.49	2.27	2.45
Ash	4.71	4.61	4.62	4.62	4.86
ABC-4, meq/kg	383	356	287	373	333

^*a*^Phase 1 diets were fed according to a feed budget of 1.8 kg/pig.

^*b*^AX3 Digest; Protekta; Newport Beach, CA.

^*c*^MEPRO; Prairie Aquatech; Brookings, SD.

^*d*^AV-E Digest; XFE Products; Des Moines, IA.

^*e*^Appetein; APC Inc., Ankeny, IA.

^*f*^Provided per kg of diet: 10,472 IU vitamin A; 83 IU vitamin E; 4 mg vitamin K; 0.03 mg vitamin B12; 40 mg niacin; 30 mg pantothenic acid; 8 mg riboflavin; 100 mg iron; 16 mg copper; 0.5 mg iodine; 0.30 mg selenium. Vitamin-trace mineral premix contained Optiphos PLUS (Huvepharma Inc.; Peachtree City, Georgia) which provided an estimated release of 0.12% STTD P with 647 FTU/kg.

^*g*^ Zinpro, Eden Prairie, MN.

^*h*^Calculated ABC-4 values were determined based on the analyzed experimental protein sources and all other ingredient ABC-4 values were obtained from Stas et al. (2024).

**Table 4.** Phase 2 diet composition (as-fed basis), experiment 1^*a*^

		Specialty soy protein concentrate^*b*^		Microbially enhanced soybean meal^*c*^	
Item	Control	Replacing poultry meal	Replacing poultry meal and blood plasma	Replacing poultry meal	Replacing poultry meal and blood plasma
Ingredients, %					
Corn	52.72	54.96	53.03	54.77	52.71
Soybean meal (46.8% CP)	22.76	22.76	22.60	22.91	22.87
Cereal blend	5.00	5.00	5.00	5.00	5.00
Poultry meal^*d*^	9.50	—	—	—	—
Specialty soy protein concentrate^*b*^	—	6.00	10.75	—	—
Microbially enhanced soybean meal^*c*^	—	—	—	6.00	10.75
Spray-dried blood plasma^*e*^	2.75	2.75	—	2.75	—
Crystalline lactose	1.38	1.38	1.38	1.38	1.38
Fat blend	2.00	2.00	2.00	2.00	2.00
Beef tallow	0.50	0.50	0.50	0.50	0.50
Limestone	0.53	0.75	0.55	0.83	0.70
Dicalcium phosphate (19% P)	0.23	1.05	1.30	0.95	1.10
Salt	0.18	0.43	0.58	0.50	0.70
L-Lys	0.49	0.49	0.49	0.49	0.49
DL-Met	0.24	0.25	0.24	0.25	0.24
L-Thr	0.27	0.27	0.26	0.27	0.25
L-Trp	0.09	0.08	0.08	0.07	0.07
L-Val	0.16	0.16	0.13	0.16	0.13
L-Ile	0.10	0.07	—	0.07	—
Copper chloride	0.04	0.04	0.04	0.04	0.04
Choline chloride	0.03	0.03	0.03	0.03	0.03
Zinc oxide	0.28	0.28	0.28	0.28	0.28
Vitamin E	0.01	0.01	0.01	0.01	0.01
Vitamin-trace mineral premix^*f*^	0.25	0.25	0.25	0.25	0.25
Benzoic acid	0.50	0.50	0.50	0.50	0.50
Organic Mn (20.0%)^*g*^	0.02	0.02	0.02	0.02	0.02
Total	100	100	100	100	100

Item	Control	Specialty soy protein concentrate^*b*^		Microbially enhanced soybean meal^*c*^	
		Replacing poultry meal	Replacing poultry meal and blood plasma	Replacing poultry meal	Replacing poultry meal and blood plasma
SID amino acids, %					
Lys	1.42	1.42	1.42	1.42	1.42
Ile:Lys	58	57	58	59	60
Leu:Lys	103	106	109	107	110
Met:Lys	36	35	36	35	36
Met and Cys:Lys	56	56	56	56	56
Thr:Lys	66	66	66	66	66
Trp:Lys	22.3	22.3	22.3	22.2	22.4
Val:Lys	71	71	71	71	71
His:Lys	33	34	34	34	34
Total Lys, %	1.61	1.58	1.57	1.58	1.58
NE NRC, kcal/kg	2,414	2,493	2,474	2,485	2,458
SID Lys:NE, g/Mcal	5.88	5.70	5.74	5.72	5.77
CP, %	22.6	22.0	22.8	22.2	23.1
Ca, %	0.64	0.64	0.64	0.64	0.64
P, %	0.56	0.58	0.61	0.55	0.56
STTD P, %	0.43	0.43	0.43	0.43	0.43
ABC-4, meq/kg^*h*^	474	442	386	461	424
Analyzed values, %					
DM	88.47	88.03	88.23	88.48	88.65
CP	21.33	22.87	22.44	22.40	22.95
Crude fat	4.13	3.51	2.88	3.21	2.93
Crude fiber	1.87	2.29	2.38	2.13	2.22
Ash	4.90	4.61	4.91	4.85	5.02
ABC-4, meq/kg	413	387	340	400	377

^*a*^Phase 2 diets were fed according to a feed budget of 5.4 kg/pig.

^*b*^AX3 Digest; Protekta; Newport Beach, CA.

^*c*^MEPRO; Prairie Aquatech; Brookings, SD.

^*d*^AV-E Digest; XFE Products; Des Moines, IA.

^*e*^Appetein; APC Inc., Ankeny, IA.

^*f*^Provided per kg of diet: 10,472 IU vitamin A; 83 IU vitamin E; 4 mg vitamin K; 0.03 mg vitamin B12; 40 mg niacin; 30 mg pantothenic acid; 8 mg riboflavin; 100 mg iron; 16 mg copper; 0.5 mg iodine; 0.30 mg selenium. Vitamin-trace mineral premix contained Optiphos PLUS (Huvepharma Inc.; Peachtree City, Georgia) which provided an estimated release of 0.12% STTD P with 647 FTU/kg.

^*g*^Zinpro, Eden Prairie, MN.

^*h*^Calculated ABC-4 values were based on analyzed experimental protein sources and all other ingredient ABC-4 values were obtained from Stas et al. (2024).

### Experiment 2

#### Animal and treatment structure.

The objective of experiment 2 was to determine the effects of varying the ABC-4 diet levels by using different specialty soy protein sources with or without pharmacological levels of Zn on nursery pig performance. A total of 1,057 pigs (PIC 800 × [Fast LW × PIC L02], initially 6.2 kg) were used in a 42-d nursery trial. Pigs were weaned at approximately 21 d of age, and pens of pigs were randomly assigned to one of the four dietary treatments based on initial BW. There were 22 pigs per pen and 12 replications per treatment.

Four dietary treatments were arranged in a 2 × 2 factorial with the main effects of low or high ABC-4 and pharmacological levels of Zn from ZnO (105 or 2,000 mg/kg) fed in two phases (Tables 5 and 6). The low ABC-4 diets were formulated with 13.0% and 10.75% SSPC in phases 1 and 2, respectively. The high ABC-4 diets were formulated with 15.85% and 13.15% ESBM in phases 1 and 2, respectively, replacing the SSPC on an equal SID Lys basis. SSPC and ESBM were measured to have an ABC-4 value of −13 and 753 meq/kg, respectively. The low ABC-4 diets without pharmacological levels of Zn were formulated to contain 150 and 200 meq/kg in phases 1 and 2, respectively. Replacing SSPC with ESBM increased the ABC-4 of the diet by approximately 104 to 127 meq/kg. The addition of ZnO increased the ABC-4 of the diet by approximately 60 to 65 meq/kg. Diets without ZnO contained 105 mg/kg of Zn that was added individually because the vitamin–trace mineral premix did not contain any Zn.

**Table 5.** Phase 1 diet composition (as-fed basis), experiment 2^*a*^

	No ZnO		Added ZnO^*b*^	
Item	Low ABC-4^*c*^	High ABC-4^*c*^	Low ABC-4^*c*^	High ABC-4^*c*^
Ingredients, %				
Corn	42.81	39.85	42.51	39.55
Soybean meal (46.8% CP)	18.58	18.58	18.58	18.58
Cereal blend	13.10	13.10	13.10	13.10
Specialty soy protein concentrate^*d*^	13.00	—	13.00	—
Enzymatically treated soybean meal^*e*^	—	15.85	—	15.85
Oat groats	3.75	3.75	3.75	3.75
Crystalline lactose	1.38	1.38	1.38	1.38
Fumaric acid	1.27	1.27	1.27	1.27
Fat blend	2.00	2.00	2.00	2.00
Beef tallow	0.50	0.50	0.50	0.50
Limestone	0.32	0.40	0.32	0.40
Dicalcium phosphate (19% P)	1.40	1.20	1.40	1.20
Salt	0.45	0.63	0.45	0.63
L-Lys	0.42	0.42	0.42	0.42
DL-Met	0.22	0.24	0.22	0.24
L-Thr	0.26	0.28	0.26	0.28
L-Trp	0.07	0.06	0.07	0.06
L-Val	0.06	0.08	0.06	0.08
Copper chloride	0.04	0.04	0.04	0.04
Choline chloride	0.03	0.03	0.03	0.03
Zinc oxide	—	—	0.30	0.30
Organic Zn (21.0%)^*f*^	0.05	0.05	0.05	0.05
Vitamin E	0.05	0.05	0.05	0.05
Vitamin-trace mineral premix^*g*^	0.25	0.25	0.25	0.25
Organic Mn (20.0%)^*f*^	0.02	0.02	0.02	0.02
Total	100	100	100	100

	No ZnO		Added ZnO^*b*^	
Item	Low ABC-4^*c*^	High ABC-4^*c*^	Low ABC-4^*c*^	High ABC-4^*c*^
SID amino acids, %				
Lys	1.36	1.36	1.36	1.36
Ile:Lys	61	60	61	60
Leu:Lys	113	108	113	108
Met:Lys	36	37	36	37
Met and Cys:Lys	56	56	56	56
Thr:Lys	66	66	66	66
Trp:Lys	22.3	22.2	22.3	22.2
Val:Lys	70	70	70	70
His:Lys	36	36	36	36
Total Lys, %	1.51	1.52	1.51	1.52
NE, kcal/kg	2,368	2,350	3,359	2,341
SID Lys:NE, g/Mcal	5.75	5.79	5.76	5.81
CP, %	22.5	22.4	22.5	22.4
Ca, %	0.58	0.59	0.58	0.59
P, %	0.61	0.62	0.61	0.62
STTD P, %	0.46	0.46	0.46	0.46
ABC-4, meq/kg	150	277	215	342
Analyzed values, %				
DM	88.84	88.78	89.02	89.01
CP	22.01	22.38	22.29	23.23
Crude fat	3.74	4.06	3.87	3.73
Crude fiber	2.52	2.52	2.45	2.28
Ash	4.89	5.35	4.94	5.52
Zn	254	158	1,710	2,167
ABC-4, meq/kg	143	267	167	290

^*a*^Phase 1 diets were fed according to a feed budget of 1.8 kg/pig.

^*b*^Diets were formulated to contain 2,265 mg/kg Zn from ZnO.

^*c*^ABC at a pH of 4. The amount of hydrochloric acid required to achieve a stable pH of 4 for 1 kg of diet.

^*d*^AX3 Digest; Protekta; Newport Beach, CA.

^*e*^HP 300; Hamlet Protein; Findlay, OH.

^*f*^Zinpro, Eden Prairie, MN.

^*g*^Provided per kg of diet: 10,472 IU vitamin A; 83 IU vitamin E; 4 mg vitamin K; 0.03 mg vitamin B12; 40 mg niacin; 30 mg pantothenic acid; 8 mg riboflavin; 100 mg iron; 16 mg copper; 0.5 mg iodine; 0.30 mg selenium. Vitamin-trace mineral premix contained Optiphos PLUS (Huvepharma Inc.; Peachtree City, Georgia) which provided an estimated release of 0.12% STTD P with 647 FTU/kg.

**Table 6.** Phase 2 diet composition (as-fed basis), experiment 2^*a*^

	No ZnO		Added ZnO^*b*^	
Item	Low ABC-4^*c*^	High ABC-4^*c*^	Low ABC-4^*c*^	High ABC-4^*c*^
Ingredients, %				
Corn	52.32	49.87	52.04	49.59
Soybean meal (46.8% CP)	22.65	22.65	22.65	22.65
Cereal blend	5.00	5.00	5.00	5.00
Specialty soy protein concentrate^*d*^	10.75	—	10.75	—
Enzymatically treated soybean meal^*e*^	—	13.15	—	13.15
Crystalline lactose	1.38	1.38	1.38	1.38
Fumaric acid	1.38	1.38	1.38	1.38
Fat blend	2.00	2.00	2.00	2.00
Beef tallow	0.50	0.50	0.50	0.50
Limestone	0.56	0.63	0.56	0.63
Dicalcium phosphate (19% P)	1.30	1.13	1.30	1.13
Salt	0.58	0.70	0.58	0.70
L-Lys	0.49	0.49	0.49	0.49
DL-Met	0.25	0.26	0.25	0.26
L-Thr	0.26	0.27	0.26	0.27
L-Trp	0.08	0.08	0.08	0.08
L-Val	0.13	0.14	0.13	0.14
Copper chloride	0.04	0.04	0.04	0.04
Choline chloride	0.03	0.03	0.03	0.03
Zinc oxide	—	—	0.28	0.28
Organic Zn (21.0%)^*f*^	0.05	0.05	0.05	0.05
Vitamin E	0.01	0.01	0.01	0.01
Vitamin-trace mineral premix^*g*^	0.25	0.25	0.25	0.25
Organic Mn (20.0%)^*f*^	0.02	0.02	0.02	0.02
Total	100	100	100	100

	No ZnO		Added ZnO^*b*^	
Item	Low ABC-4^*c*^	High ABC-4^*c*^	Low ABC-4^*c*^	High ABC-4^*c*^
SID amino acids, %				
Lys	1.42	1.42	1.42	1.42
Ile:Lys	58	57	58	57
Leu:Lys	109	106	109	105
Met:Lys	37	37	37	37
Met and Cys:Lys	56	56	56	56
Thr:Lys	63	63	63	63
Trp:Lys	22.3	22.2	22.3	22.2
Val:Lys	71	71	71	71
His:Lys	34	34	34	34
Total Lys, %	1.57	1.58	1.57	1.58
NE, kcal/kg	2,456	2,443	2,449	2,434
SID Lys:NE, g/Mcal	5.78	5.82	5.80	5.84
CP, %	22.7	22.6	22.7	22.6
Ca, %	0.64	0.64	0.64	0.64
P, %	0.61	0.61	0.61	0.61
STTD P, %	0.46	0.46	0.46	0.46
ABC-4, meq/kg	200	304	261	365
Analyzed values, %				
DM	89.05	89.02	89.20	89.91
CP	22.03	22.66	22.35	22.30
Crude fat	3.88	4.08	3.94	3.90
Crude fiber	2.16	2.00	2.14	2.18
Ash	4.86	5.45	5.49	5.55
Zn	321	255	1,743	1,805
ABC-4, meq/kg	170	260	197	293

^*a*^Phase 2 diets were fed according to a feed budget of 5.4 kg/pig.

^*b*^Diets were formulated to contain 2,121 mg/kg Zn from ZnO.

^*c*^ABC at a pH of 4. The amount of hydrochloric acid required to achieve a stable pH of 4 for 1 kg of diet.

^*d*^AX3 Digest; Protekta; Newport Beach, CA.

^*e*^HP 300; Hamlet Protein; Findlay, OH.

^*f*^Zinpro, Eden Prairie, MN.

^*g*^Provided per kg of diet: 10,472 IU vitamin A; 83 IU vitamin E; 4 mg vitamin K; 0.03 mg vitamin B12; 40 mg niacin; 30 mg pantothenic acid; 8 mg riboflavin; 100 mg iron; 16 mg copper; 0.5 mg iodine; 0.30 mg selenium. Vitamin-trace mineral premix contained Optiphos PLUS (Huvepharma Inc.; Peachtree City, Georgia) which provided an estimated release of 0.12% STTD P with 647 FTU/kg.

### Experiments 1 and 2

For both experiments, pigs were fed experimental diets according to a feed budget of 1.8 and 5.4 kg/pig for phases 1 and 2, respectively. Growth performance was determined for comparison of treatments from d 0 to 7, d 7 to 21, d 0 to 21, d 21 to 42, and overall (d 0 to 42). Following phase 2 diets, all pigs were fed a common corn–soybean meal-based diet for the duration of the study. Experimental diets were formulated to contain 1.36% (phase 1) and 1.42% (phase 2) SID Lys and met or exceeded other AA relative to Lys requirement estimates established by the NRC (2012). Pens of pigs were weighed and feed disappearance was measured weekly from d 7 to 42 to determine average daily gain (**ADG**), average daily feed intake (**ADFI**), and gain-to-feed ratio (G:F). Overall growth performance was also calculated on a per-pig placed basis by factoring in the observed mortalities amongst the treatments. Overall growth performance on a per-pig placed basis was achieved by calculating BW, ADG, ADFI, and G:F based on the initial inventory at the start of each experiment. Therefore, the weight of mortalities was not included in the calculations to account for mortality loss by treatment. ADFI was not adjusted for mortality, but gain was accrued by the pigs until the time of removal which is considered within the calculation of ADG and G:F. Under the circumstances where a pig died or needed to be removed from the study due to sickness or injury, the weight of the pig, the pen they originated from, the reason for removal, and the date of removal were recorded for analysis. For experiment 2, on approximately d 15 of the trial, the research site experienced a porcine reproductive and respiratory syndrome virus (**PRRSV**) challenge. The PRRSV outbreak was confirmed by the University of Minnesota Veterinary Diagnostic Laboratory (St. Paul, MN) using real-time polymerase chain reaction of fixed lung tissue.

### Statistical analysis

In both experiments, data were analyzed as a randomized complete block design with BW serving as a blocking factor and pen as the experimental unit. Block was included in the model as a random effect. All data were analyzed using the RStudio environment [Version 4.0.2 (2020-06-22), R Core Team, R Foundation for Statistical Computing, Vienna, Austria]. For experiment 1, treatment was included in the model as a fixed effect using the lmer function. Interactive and main effects of specialty soy protein sources, replacement of PM, and replacement of SDBP were tested using pre-planned contrast statements. For experiment 2, the main effects of ABC-4 and added ZnO, as well as their interactions, were also tested using the lmer function. When a significant interaction was present, contrast statements were used to characterize the simple effects. Removals and mortality for both experiments were analyzed as a binomial using the glmer function. Results were considered significant at *P *≤ 0.05 and marginally significant at 0.05 < *P *≤ 0.10.

## RESULTS

### Experiment 1

#### ABC-4 analysis.

All analyzed ABC-4 diet values were slightly less than the calculated values. However, analyzed ABC-4 still changed relative to the experimental protein sources utilized. Analyzed values were on average 56 and 54 meq/kg lower than calculated values for phases 1 and 2, respectively.

There were no interactions observed between specialty soy protein source and specialty protein replacement strategy for any growth performance criteria or removals and mortality for the duration of the experiment (Table 7).

**Table 7.** Effects of specialty soy protein products to replace poultry meal and blood plasma in nursery diets, experiment 1^*a*^

		Specialty soy protein concentrate^*b*^		Microbially enhanced soybean meal^*c*^			*P* ^*d*^ value		
Item	Control	Replacing poultry meal	Replacing poultry meal and blood plasma	Replacing poultry meal	Replacing poultry meal and blood plasma	SEM	Soy protein source^*e*^	Replacing poultry meal with soy source^*f*^	Replacing blood plasma with soy source^*g*^
BW, kg									
d 0	6.7	6.7	6.7	6.7	6.7	0.06	0.954	0.796	0.819
d 7	6.9	7.0	7.1	7.0	7.1	0.06	0.503	0.001	0.071
d 21	10.9	11.6	11.7	11.4	11.6	0.13	0.210	<0.001	0.130
d 42	20.5	21.1	21.4	21.1	21.2	0.31	0.548	0.028	0.392
d 42 per-pig placed^*h*^	19.4	20.0	20.5	20.3	20.6	0.38	0.555	0.020	0.135
Day 0 to 7									
ADG, g	21	41	49	44	53	5.0	0.477	<0.001	0.075
ADFI, g	65	88	81	89	98	4.6	0.063	<0.001	0.789
G:F g/kg	304	463	594	444	548	51.4	0.515	0.016	0.020
Day 7 to 21									
ADG, g	286	323	326	309	320	6.1	0.098	<0.001	0.251
ADFI, g	351	389	387	382	396	6.3	0.846	<0.001	0.332
G:F, g/kg	814	831	843	810	808	8.2	<0.001	0.495	0.529
Experimental period (d 0 to 21)									
ADG, g	197	228	233	220	230	4.6	0.229	<0.001	0.097
ADFI, g	254	288	284	283	296	4.9	0.439	<0.001	0.341
G:F, g/kg	772	794	819	775	778	8.0	<0.001	0.162	0.044
Day 21 to 42									
ADG, g	451	443	457	458	456	12.3	0.370	0.964	0.428
ADFI, g	666	691	706	692	702	14.0	0.838	0.044	0.236
G:F, g/kg	676	640	646	661	649	8.9	0.079	0.002	0.735
Overall (d 0 to 42)									
ADG, g	322	334	343	337	342	6.9	0.890	0.016	0.135
ADFI, g	456	486	492	484	497	8.1	0.834	0.001	0.181
G:F, g/kg	704	686	698	695	688	6.6	0.874	0.029	0.630
Overall (d 0 to 42) per-pig placed									
ADG, g^*i*^	303	318	329	323	331	8.7	0.552	0.019	0.139
ADFI, g^*j*^	442	474	481	473	487	9.1	0.710	<0.001	0.162
G:F, g/kg	682	669	683	679	677	9.1	0.725	0.389	0.409
Removals, %^*k*^	4.5	4.2	2.7	2.7	2.0	0.94	0.245	0.376	0.231
Mortality, %^*l*^	1.1	0.9	1.3	1.3	1.1	0.58	0.851	0.988	0.851
Removals and mortality, %	5.6	5.1	4.0	4.0	3.1	1.08	0.416	0.349	0.333

^*a*^A total of 2,260 pigs (initially 6.7 ± 0.06 kg) across two facilities were used in a 42-d nursery trial. A total of five dietary treatments were used with two different specialty soy protein sources replacing poultry meal or poultry meal and spray-dried blood plasma. In the first facility, there were 20 pigs per pen and 10 pens per treatment spread across two barns. In the second facility, there were 21 pigs per pen and 12 replications per treatment. In total, there were 22 replications per treatment.

^*b*^AX3 Digest; Protekta; Newport Beach, CA.

^*c*^MEPRO; Prairie Aquatech; Brookings, SD.

^*d*^There were no soy protein source × poultry meal and spray-dried blood plasma replacement interaction observed (*P* > 0.05) throughout the duration of the study.

^*e*^Compares the mean of pigs fed diets containing specialty soy protein concentrate to those fed diets containing microbially enhanced soybean meal.

^*f*^Compares the control to the mean of each poultry meal replacement diet.

^*g*^Compares the mean of pigs fed diets with soy protein sources replacing poultry meal to the mean of pigs fed diets with soy protein sources replacing poultry meal and spray-dried blood plasma.

^*h*^BW per-pig placed = final pen ending weight/pigs placed.

^*i*^ADG per-pig placed = (final pen ending weight − initial pen weight)/(Pigs initially placed × days of trial).

^*j*^ADFI per-pig placed = (total feed intake)/(Pigs initially placed × days of trial).

^*k*^Percentage of pigs removed from original pen.

^*l*^Percentage of pigs that died or were euthanized in original pen.

When comparing soy protein sources, from d 0 to 7, pigs fed MESBM tended to have increased (*P *= 0.063) ADFI compared with pigs fed SSPC. From d 7 to 21, pigs fed SSPC tended to have greater (*P *= 0.098) ADG compared with pigs fed MESBM. From d 7 to 21 and d 0 to 21, pigs fed SSPC had increased (*P *< 0.001) G:F compared with pigs fed MESBM. From d 21 to 42, pigs previously fed MESBM meal tended to have increased (*P *= 0.079) G:F compared with pigs previously fed SSPC. Overall (d 0 to 42) and overall on a per-pig placed basis, there were no differences among pigs fed the two specialty soy protein sources.

For the comparison of soy protein sources replacing PM, from d 0 to 7, pigs fed diets containing soy protein as a replacement to PM had increased (*P *≤ 0.016) BW, ADG, ADFI, and G:F compared with those fed the control diet with PM. From d 7 to 21 and d 0 to 21, pigs fed soy protein as a replacement to PM had increased (*P *< 0.001) BW, ADG, and ADFI compared with the control diet. From d 21 to 42, pigs previously fed soy protein as a replacement to PM had increased (*P *= 0.044) ADFI compared with pigs previously fed PM. From d 21 to 42 and overall (d 0 to 42), pigs fed the control diet with PM had increased (*P *≤ 0.029) G:F compared with pigs fed soy protein replacing PM. Overall (d 0 to 42) and overall on a per-pig placed basis, pigs fed either soy protein source replacing PM had increased (*P *≤ 0.020) BW, ADG, and ADFI compared with the control diet.

For the comparison of soy protein sources replacing SDBP, from d 0 to 7, pigs fed diets with soy protein replacing SDBP tended to have increased (*P *= 0.071) BW compared with those fed diets containing SDBP. From d 0 to 7 and d 0 to 21, pigs fed diets with either soy protein source replacing SDBP tended to have increased (*P *≤ 0.097) ADG and had increased (*P *< 0.044) G:F compared with pigs fed diets with SDBP. From d 7 to 21, d 21 to 42, overall (d 0 to 42) and overall on a per-pig placed basis, there were no differences in growth performance between pigs fed diets containing SDBP and pigs fed diets with either soy protein source as a replacement to SDBP.

### Experiment 2

#### ABC-4 analysis.

All analyzed ABC-4 diet values were slightly less than the calculated values. However, analyzed ABC-4 still increased with the inclusion of ESBM and ZnO. Analyzed values were on average 8.5 and 37 meq/kg lower than calculated values in diets without ZnO in phases 1 and 2, respectively. Diets with ZnO had analyzed values on average 50 and 68 meq/kg lower than calculated values for phases 1 and 2, respectively. Stas et al. (2022) also observed lower analyzed ABC-4 diet values compared with calculated ABC-4 diet values from individual ingredients analysis. The authors also indicated the addition of ZnO contributed to the discrepancy. The average pen water pH was 7.37 and, because it was near neutral pH, it would not be expected to influence the results of the study.

For growth performance from 0 to 7, there was an ABC-4 × ZnO interaction observed (*P *= 0.036) for ADG and G:F. In diets with ZnO, pigs fed the high ABC-4 diet had greater (*P *< 0.05) ADG and a tendency for increased (*P *< 0.10) G:F compared with pigs fed the low ABC-4 diet, but when ZnO was absent, there were no differences between low and high ABC-4 diets (Table 8). From d 7 to 21, there was a tendency for an ABC-4 × ZnO interaction (*P *≤ 0.076) for ADG and G:F. When ZnO was not present, pigs fed the low ABC-4 diet had increased (*P *< 0.05) ADG and G:F compared with pigs fed the high ABC-4 diet, but when ZnO was added, there were no differences observed between pigs fed the low and high ABC-4 diets. From d 0 to 21, there was an ABC-4 × ZnO interaction observed (*P *≤ 0.026) for ADG and G:F. When ZnO was not present, pigs fed the low ABC-4 diet had increased (*P *< 0.05) ADG and G:F compared with pigs fed the high ABC-4 diet, but when ZnO was added, there were no differences between pigs fed low and high ABC-4 diets. In addition, a tendency for an ABC-4 × ZnO interaction was observed (*P *= 0.091) for ADFI. When ZnO was not present, pigs fed a low ABC-4 diet tended to have increased (*P *< 0.10) ADFI compared with pigs fed a high ABC-4 diet, but when ZnO was added, there were no differences among pigs fed low and high ABC-4 diets. There were no ABC-4 × ZnO interactions observed from d 21 to 42 or overall (d 0 to 42). However, there was an ABC-4 × ZnO interaction observed (*P *≤ 0.040) for BW and ADG overall (d 0 to 42) when data were calculated on a per-pig placed basis. When ZnO was not present, pigs fed a low ABC-4 diet had greater (*P *< 0.05) ADG and tended to have increased (*P *< 0.10) BW compared with pigs fed a high ABC-4 diet, but when ZnO was added there were no differences between pigs fed low and high ABC-4 diets.

**Table 8.** Interactive effects of ABC-4 and pharmacological ZnO on nursery pig performance^*a*^

Item	No ZnO		Added ZnO^*b*^			*P* value		
	Low ABC-4^*c*^	High ABC-4^*c*^	Low ABC-4^*c*^	High ABC-4^*c*^	SEM	ABC-4 × ZnO	ABC-4	ZnO
BW, kg								
d 0	6.2	6.2	6.2	6.2	0.11	0.196	0.775	0.324
d 7	6.3	6.3	6.5	6.6	0.11	0.303	0.228	<0.001
d 21	9.2	8.9	9.5	9.7	0.20	0.230	0.642	0.008
d 42	17.5	17.8	17.8	17.8	0.29	0.617	0.747	0.623
d 42 per-pig placed^*d*^	14.4	12.8	14.3	15.1	0.60	0.040	0.466	0.050
Day 0 to 7								
ADG, g^*e*^	23	14	39	59	6.8	0.036	0.415	<0.001
ADFI, g	72	71	80	85	4.2	0.461	0.671	0.012
G:F, g/kg	318	163	459	702	92.9	0.036	0.634	0.001
Day 7 to 21								
ADG, g^*f*^	191	145	193	196	13.1	0.063	0.106	0.043
ADFI, g	268	233	271	276	12.1	0.102	0.223	0.062
G:F, g/kg^*f*^	702	607	709	708	26.3	0.076	0.070	0.045
Experimental period (d 0 to 21)								
ADG, g^*f*^	133	98	140	149	9.5	0.026	0.175	0.004
ADFI, g	200	176	205	210	8.7	0.091	0.247	0.027
G:F, g/kg^*f*^	655	543	676	707	29.3	0.017	0.166	0.003
Day 21 to 42								
ADG, g	374	394	372	370	10.9	0.306	0.409	0.218
ADFI, g	521	542	528	531	11.1	0.397	0.299	0.830
G:F, g/kg	716	727	703	697	10.0	0.408	0.827	0.036
Overall (d 0 to 42)								
ADG, g	246	229	247	254	8.5	0.152	0.563	0.124
ADFI, g	351	338	354	363	8.7	0.202	0.788	0.106
G:F, g/kg	699	673	696	701	12.1	0.192	0.367	0.308
Overall (d 0 to 42) per-pig placed								
ADG, g^*f*^^,^^*g*^	201	158	193	213	13.4	0.024	0.394	0.080
ADFI, g^*h*^	308	291	327	320	12.4	0.640	0.307	0.047
G:F, g/kg	643	583	587	672	0.063	0.240	0.835	0.789
Experimental period (d 0 to 21)^*i*^								
Removals, %^*j*^	3.3	6.2	3.4	3.0	1.55	0.239	0.417	0.285
Mortality, %^*f*^^,^^*k*^	5.5	11.2	7.3	6.6	2.23	0.064	0.167	0.557
Removals and mortality, %^*f*^	9.0	17.7	11.0	9.8	2.49	0.025	0.102	0.241
Day 21 to 42								
Removals, %^*j*^	2.3	4.5	2.6	0.0	1.47	0.999	0.999	0.999
Mortality, %^*k*^	5.9	7.5	7.2	5.5	1.87	0.301	0.950	0.822
Removals and mortality, %	8.1	12.0	9.8	5.4	2.37	0.029	0.690	0.175
Overall (d 0 to 42)								
Removals, %^*j*^	5.3	9.9	5.7	3.0	1.92	0.021	0.986	0.037
Mortality, %^*k*^	11.1	17.8	14.0	11.7	2.46	0.043	0.354	0.554
Removals and mortality, %^*f*^	16.5	27.7	19.7	14.8	2.88	0.002	0.327	0.076

^*a*^A total of 1,057 pigs (initial BW of 6.2 ± 0.11 kg) were used in a 42-d nursery trial. A total of 4 dietary treatments were arranged in a 2 × 2 factorial design consisting of low or high ABC-4 of the diet and the absence or presence of ZnO. There were 22 pigs per pen and 12 replications per treatment.

^*b*^Diets contained 2,265 and 2,121 mg/kg of Zn from ZnO in phases 1 and 2, respectively.

^*c*^The low ABC-4 diets were formulated with a specialty soy protein concentrate (AX3 Digest; Protekta; Newport Beach, CA) and the high ABC-4 diets were formulated with enzymatically treated soybean meal (HP 300; Hamlet Protein; Findlay, OH). The low ABC-4 diet without ZnO was formulated to a value of 150 and 200 meq/kg in phases 1 and 2, respectively. The addition of ZnO increased the ABC-4 of the diet by approximately 60 to 65 meq/kg. Replacing specialty soy protein concentrate with enzymatically treated soybean meal increased the ABC-4 of the diet by 104 to 127 meq/kg.

^*d*^BW per-pig placed = final pen ending weight ÷ pigs placed.

^*e*^ABC-4 effect without ZnO, *P *> 0.10. ABC-4 effect with ZnO, *P *< 0.05.

^*f*^ABC-4 effect without ZnO, *P *< 0.05. ABC-4 effect with ZnO, *P *> 0.10.

^*g*^ ADG per-pig placed = (final pen ending weight − initial pen weight)/(Pigs initially placed × days of trial).

^*h*^ADFI per-pig placed = (total feed intake)/(Pigs initially placed × days of trial).

^*i*^The research site experienced a PRRS disease challenge in approximately d 15 of the trials.

^*j*^Percentage of pigs that were removed into a hospital pen.

^*k*^Percentage of pigs that died or euthanized in original pen or hospital pen after being removed.

For ZnO main effects, from d 0 to 7, pigs fed diets containing ZnO had increased (*P *≤ 0.012) BW and ADFI compared with pigs fed diets without ZnO. From d 7 to 21, pigs fed diets containing ZnO tended to have increased (*P *= 0.062) ADFI compared with pigs fed diets without ZnO. From d 21 to 42, pigs previously fed diets without ZnO had increased (*P *= 0.036) G:F compared with pigs fed diets with added ZnO.

From d 0 to 21, there was an ABC-4 × ZnO interaction (*P *= 0.025) for total removals and mortalities and a tendency for an interaction (*P *= 0.064) for mortalities. When ZnO was not present, pigs fed a low ABC-4 diet had decreased (*P *< 0.05) mortalities and total removals and mortalities compared with pigs fed a high ABC-4 diet, but when ZnO was added there were no differences between pigs fed low and high ABC-4 diets. From d 21 to 42, there was an ABC-4 × ZnO interaction observed (*P *= 0.029) for total removals and mortalities. In diets with ZnO, pigs previously fed a high ABC-4 diet had a tendency for decreased (*P *< 0.10) total removals and mortalities compared with a low ABC-4 diet, but when ZnO was absent there were no differences between low and high ABC-4 diets. Overall (d 0 to 42), there was an ABC-4 × ZnO interaction observed (*P *≤ 0.043) for mortalities, and total removals and mortalities. When ZnO was not present, pigs fed a low ABC-4 diet had decreased (*P *< 0.05) total removals and mortalities and tended to have decreased (*P *< 0.10) mortalities compared with pigs fed a high ABC-4 diet, but when ZnO was added there were no differences between low and high ABC-4 diets.

## DISCUSSION

Further-processed soy proteins are widely used in the swine industry because of their decreased allergenic components relative to conventional soybean meal and their cost competitiveness compared with animal protein sources (Long et al., 2021). However, there are limited data suggesting which soy protein source will optimize performance and health status compared with others. For example, Ma et al. (2019) observed weanling pigs fed ESBM had improved performance compared with soy protein concentrate, whereas the opposite response was observed in a study conducted by Yang et al. (2007). Additionally, Yuan et al. (2017) observed no differences in growth comparing two different specialty soy protein sources. SSPC and MESBM may provide an added benefit compared with other specialty soy protein sources, such as ESBM, because of their low ABC-4 characteristics. However, there is limited research available evaluating this comparison. Microbially enhanced soybean meal has been observed to have comparable performance to fish meal in weanling pig diets (Sinn et al., 2017). Further evaluation of these low ABC-4 soy protein sources is warranted to understand whether their low ABC-4 characteristics provide an additional opportunity compared with higher ABC-4 animal proteins and other specialty soy protein sources in weanling pig diets.

ABC at a pH of 4 is the amount of hydrochloric acid required to achieve a stable pH of 4 for 1 kg of ingredient. Stas et al. (2022) observed common protein sources used for weanling pigs range widely in their ABC-4 values from –13 to 1,380 meq/kg. Among those protein sources, SSPC and MESBM had the lowest ABC-4 among protein sources. In the current study, SSPC and MESBM were observed to have an ABC-4 of –13 and 107 meq/kg, respectively. Whereas SDBP and PM were analyzed to have an ABC-4 of 713 and 1,007 meq/kg, respectively. Analyzed samples of PM and SDBP had higher levels of ash than SSPC and MESBM which contributes to the large difference in ABC-4 from the presence of cationic minerals (Jasaitis et al., 1987; Lawlor et al., 2005). Although animal protein sources are generally considered higher quality than vegetable proteins, SSPC and MESBM provide an advantage in having a lower ABC-4.

In experiment 1, SSPC and MESBM were evaluated as a replacement to PM or SDBP in early nursery diets. Rojas and Stein (2013) observed pigs fed diets containing fermented soybean meal had comparable performance with diets containing PM. In the current study, pigs fed soy proteins as a replacement to PM exhibited increased ADG and ADFI. The difference in growth rate observed in this study was driven by a reduction in ADFI of the diets containing PM. Therefore, the soy protein sources used in this study likely had improved palatability compared with PM. Previous research has suggested that the response to PM is variable depending on the source and quality which can affect its palatability (Keegan et al., 2004). PM palatability can be influenced depending on the processing plant it originates from and the proportions of heads, feet, and inedible viscera that comprise the meal after rendering (Babatunde et al., 2021). SDBP is considered a highly palatable and digestible protein source for weanling pigs that typically yields a more consistent response than PM (Pérez-Bosque et al., 2016). Deng et al. (2022) observed a linear reduction in BW, ADG, ADFI, and G:F when replacing PM, fish meal, and SDBP with a soy protein concentrate. Similarly, Espinosa et al. (2022) observed decreased ADG and G:F when soy protein concentrate replaced SDBP. The authors also observed looser stool associated with the pigs fed soy protein concentrate compared with SDBP. However, the soy proteins used by Deng et al. (2022) and Espinosa et al. (2022) originated from different manufacturers than the low acid-binding soy proteins used in experiment 1. In the current study, replacing SDBP with low ABC-4 soy proteins numerically increased ADG and significantly improved G:F during the experimental period. The results of these studies indicate that a low ABC soy protein may be used to replace high-quality animal proteins without diminishing performance which previously has not been observed.

When directly comparing the ABC-4 between SSPC and MESBM, SSPC is 120 meq/kg lower in its ABC-4 value than MESBM. This difference in ABC-4 could explain the results of experiment 1. Pigs fed SSPC had improved G:F during the experimental period compared with MESBM. It can be theorized that SSPC was able to maintain a more acidic environment in the stomach of weanling pigs than MESBM which led to the improvement in nutrient utilization. Ruckman et al. (2020) also observed differences in weanling pig G:F when feeding different specialty soy protein sources. While the authors did not measure ABC-4 of the specialty soy protein sources, the specialty soy protein source with the lowest ash content had the most improved G:F from d 0 to 14 post-weaning. Ash content of ingredients has been observed to be positively correlated with ABC-4 values because of increased concentrations of cationic minerals such as Ca and Mg (Jasaitis et al., 1987; Lawlor et al., 2005). While low ABC-4 characteristics of soy protein sources may explain the improvements in G:F, experiment 1 diets were not formulated to a specific ABC-4 level which was our intention with formulation in experiment 2.

In experiment 2, SSPC was used to target an ABC-4 level of 150 meq/kg from d 0 to 7 post-weaning followed by 200 meq/kg from d 7 to 21 post-weaning. These ABC-4 targets were based on European recommendations to improve performance and fecal consistency (Gilles Langeoire, personal communication, November 4, 2022). Lawlor et al. (2006) observed pigs fed an ABC-4 level of 180 meq/kg for 28 d post-weaning resulted in the most improved growth performance compared with higher ABC-4 levels. In the current study, the ABC-4 level of the diet was adjusted between the two dietary phases to coincide with the developing gastrointestinal tract of weanling pigs. Additionally, phase feeding is a common practice in the swine industry to match the nutrient requirements and digestive capabilities of weanling pigs. In contrast to experiment 1, ESBM was used as the other soy protein source because of its high ABC-4 value. Replacing SSPC with ESBM on an SID Lys basis increased the ABC-4 level of the diet by 127 and 104 meq/kg in phases 1 and 2, respectively. All diets contained 105 mg/kg of Zn provided by an organic Zn source. The use of pharmacological levels of Zn from ZnO also further increased the ABC-4 of the diet because of the high ABC-4 value for ZnO. Although a high ABC-4 is thought to be more detrimental to early nursery pigs, ZnO has multiple modes of action to overcome the increase in ABC-4. Zinc oxide reduces pro-inflammatory cytokines, increases anti-inflammatory cytokines, maintains healthy morphology of the gastrointestinal tract, prevents attachment of pathogenic bacteria, and reduces intestinal permeability (Huang et al., 1999; Stark et al., 2013; Peng et al., 2020). However, the use of pharmacological levels of Zn has been banned in the European Union and Canada. Therefore, low ABC-4 formulation may be a potential alternative to pharmacological levels of Zn.

The results of experiment 2 indicate that utilizing SSPC to achieve a low ABC-4 level can improve performance and reduce mortality compared with a high ABC-4 soy protein. The PRRSV disease challenge was identified on d 15 of the experiment. The health challenge in combination with a commercial setting allowed the opportunity to observe removal and mortality differences that are typically not able to be observed in smaller studies. The use of SSPC to achieve low ABC-4 diets not only improved ADG and G:F, but also led to fewer occurrences of morbidity and mortality under the PRRSV disease challenge in diets without pharmacological levels of Zn from ZnO. The low ABC-4 diets also led to similar performance as the diets containing ZnO. Therefore, low ABC-4 formulation strategies may be used as a replacement to pharmacological levels of Zn from ZnO to maintain performance and health status. Strategies such as low CP diets or high fiber diets have been suggested as alternatives to ZnO, but these strategies are generally associated with a reduction in performance (Yan et al., 2017; Batson et al., 2021; Bonetti et al., 2021). To the best of our knowledge, this study is one of the first to illustrate the effect low acid-binding soy proteins have on nursery pig performance and mortality in a commercial environment. There is extensive research evaluating different specialty soy protein sources, but most of which do not evaluate differences in ABC-4 and how that can influence the response in young pigs. While low ABC-4 specialty soy proteins aid in lowering the overall diet ABC-4 level, other diet formulation strategies can be used as well. In addition to the protein sources used in the current study, other low ABC-4 dietary formulation strategies were incorporated to achieve the low ABC-4 levels utilized in experiment 2 of 150 and 200 meq/kg in phases 1 and 2, respectively. These strategies included the use of crystalline lactose, lower diet Ca levels, and use of dietary acidifiers which provide additional examples to lower diet ABC-4. The current study observed a benefit to an ABC-4 level of 150 and 200 meq/kg compared with 267 and 304 meq/kg from d 0 to 7 and 7 to 21 post-weaning, respectively. However, the current study only evaluated two ABC-4 levels, and more research is needed to understand what the optimal ABC-4 level is of a complete diet for a specific age or weight range on a weanling pig.

In summary, the results of experiment 1 indicate that using low acid-binding soy protein can improve the performance of nursery pigs compared with animal protein sources. The results of experiment 2 indicate that low acid-binding soy proteins can be used to target a low ABC-4 level to improve performance and reduce removals and mortality in diets not containing pharmacological levels of Zn from ZnO. Low ABC specialty soy protein sources offer an advantage in weanling pig diets as a method to maintain an acidic gastric environment in the stomach of the newly weaned pig to improve performance and maintain health.
